# Learning Automata-based Misinformation Mitigation via Hawkes Processes

**DOI:** 10.1007/s10796-020-10102-8

**Published:** 2021-02-12

**Authors:** Ahmed Abouzeid, Ole-Christoffer Granmo, Christian Webersik, Morten Goodwin

**Affiliations:** 1 Centre for Artificial Intelligence Research, University of Agder, Grimstad, Norway; 2 Center for Integrated Emergency Management, University of Agder, Kristiansand, Norway

**Keywords:** Learning automata, Stochastic optimization, Social media *Misinformation*, Crisis mitigation, Hawkes processes

## Abstract

Mitigating *misinformation* on social media is an unresolved challenge, particularly because of the complexity of information dissemination. To this end, Multivariate Hawkes Processes (MHP) have become a fundamental tool because they model social network dynamics, which facilitates execution and evaluation of mitigation policies. In this paper, we propose a novel light-weight intervention-based *misinformation* mitigation framework using decentralized Learning Automata (LA) to control the MHP. Each automaton is associated with a single user and learns to what degree that user should be involved in the mitigation strategy by interacting with a corresponding MHP, and performing a joint random walk over the state space. We use three *Twitter* datasets to evaluate our approach, one of them being a new COVID-19 dataset provided in this paper. Our approach shows fast convergence and increased valid information exposure. These results persisted independently of network structure, including networks with central nodes, where the latter could be the root of *misinformation*. Further, the LA obtained these results in a decentralized manner, facilitating distributed deployment in real-life scenarios.

## Introduction

The spread of *misinformation* on social media can have critical consequences during a crisis. Whether the crisis is a disaster, political struggle, terrorist attack, natural hazard, or a pandemic, misleading information such as rumors and false alarm can impede or endanger a successful outcome, such as effective response to a natural hazard. According to a recent study (Bradshaw and Howard 2017), at least 50*%* of the world’s countries suffer from organized political manipulation campaigns over social media. Other examples of the damaging effect of *misinformation* circulated over social media includes the Ebola outbreak in West Africa (Jin et al. 2014), which was believed to be three times more worse than the previous Ebola outbreaks. Nowadays, with a more connected world, the impact of *misinformation*^1^ is getting more severe, even becoming a global threat. For instance, the propagated climate change denying content. There is thus an increasing interest among researchers, and society in general, in finding solutions for combating *misinformation*.

There are two main strategies for combating social media *misinformation* (Sharma et al. 2019). Some research focus on classifying fake news, rumors, or fake accounts such as social bots, cyborgs, and trolls. Usually, such an approach is referred to as fake news, *misinformation*, or rumor identification. To this end, several solutions have been proposed. For instance, opinion-based or content-based solutions (Shu et al. 2017) can be used to classify fake news based on textual content. Another approach is to mitigate actively, through proactive intervention (Farajtabar et al. 2017), or after *misinformation* already is spreading throughout the social network.

Large-scale manipulation carried out across social media during political events is one of the greatest threats to social justice (Bradshaw and Howard 2017). So-called cyber armies like the Russian Trolls attack on the U.S.A 2016 presidential elections is a well-known example (Zannettou et al. 2019). To the best of our knowledge, most of the attempts to automate the detection of such malicious accounts are not real-time. Furthermore, these cyber armies change their behavior over time, and each context would often require a new model. That is, cyber armies acting in different societies and cultures will have their own linguistic- and behavioral patterns (Shu et al. 2019). This diversity makes it difficult to build all-encompassing linguistic models, leading to sub-optimal performance. Too high false negative rate leads to undetected *misinformation* attempts while too high false positive rates can be ethically problematic because accounts or content may be falsely flagged as malicious.

Our work presented in this paper addresses the above challenges by mitigating *misinformation* attempts by countering *misinformation* with rectifying information. That is, we seek to reducing the harmful effects of *misinformation* through a targeted real-news campaign. We propose an approach to single out candidate users for real-news, so as to maximize the remedying effect of injecting real-news into the social network. A real news campaign can be viewed as a counteraction to the *misinformation* process over the network. That means selecting some users such that by sharing a suggested content through them, a maximal influence would occur on all the other network users, as the latter would became more exposed to valid information.

### Hawkes Simulation

Since real-time intervention with social media platforms is not feasible for experimentation purposes, we simulate the process of information diffusion by employing Hawkes Processes (Rizoiu et al. 2017; Laub et al. 2015), as applied in Farajtabar et al. (2017) and Goindani and Neville (2019). Hawkes Processes are point processes which can model the arrival or occurrence of events, indexed by time or location. There is a range of application domains that fall into such a model. For example, in finance, a Hawkes process can describe how a buy or sell transaction on the stock market (an event) influences future stock prices and transaction volume. Similarly, in geophysics, a Hawkes process can capture how an earthquake event influences the likelihood of another earthquake event happening as an after effect. For social media, we consider content such as tweets or Facebook posts as events, that have at least time-associated indices. The introduction of new content may trigger a chain of new content, for instance through retweeting, sharing, replying, and quoting.

For all of the above example processes, a Hawkes process is particularly suitable because it is a self-exciting point process where the arrival of an event is dependant on the history of all other relevant events. In this paper, we use Hawkes processes to modeling each user, so that we can simulate different user behaviors and social network dynamics, including the effect of mitigation.

Hawkes processes are random and non-linear, suitable for capturing the unpredictable and intricate nature of social media dynamics. Optimizing mitigation effects thus becomes a challenging problem, involving spatio-temporal reasoning. Furthermore, the randomness, uncertainty and incomplete information on real-life social media aggravates the difficulty of finding a global or a local minimum. We therefore Therefore, we propose a novel LA architecture in this paper, designed to operate in stochastic and unknown environments.

### Problem Statement

Let us consider a scenario where a certain amount of *misinformation* is circulating in a social network. The *misinformation* is affecting different users to varying degree, depending on the mix of correct information and misinformation facing each user. We thus define the impact of misinformation for a single user by degree of exposure, relative to correct information (Farajtabar et al. 2017). Similarly, the overall influence of misleading information on the whole network could be measured by the average exposure on all users. Since social media events are typical spatio-temporal, these measures should be quantified and reconsidered over different time stages as well.

In order to mitigate the spread of false content, we can apply an intervention-based strategy to increase the amount of valid information against malicious content, or at least to obtain a balance between the impact of false and true content on the network. The amount of either false and accurate information could be viewed as a counts. Such count represents how much each type of content was generated on the network by each user and at a specific time.

Let *A* be an adjacency matrix indicating an explicit influence. Let *A*_*ij*_ = 1 if there is a directed edge or an influence indicating that user *i* follows user *j* or quotes (with agreement) content from *j*, and *A*_*ij*_ = 0 if not. For a realization of *r* time steps {*t*_0_, *t*_1_, ... *t*_*r*_}, let $F^{t_{r}}$, and $T^{t_{r}}$ be the impacts of false- and true content exposure prior to and including time step *t*_*r*_, respectively. Hence, the impact of both false and true content on user *i* till the time stage *t*_*r*_ can be calculated as per (1), and (2), respectively.

1 $$  F^{t_{r}}_{i}= \sum\limits^{t_{r}}_{s=0} \sum\limits^{n}_{j=1} A_{ij} \cdot F^{t_{s}}_{j} $$

2 $$  T^{t_{r}}_{i}= \sum\limits^{t_{r}}_{s=0} \sum\limits^{n}_{j=1} A_{ij} \cdot T^{t_{s}}_{j} $$

The outer summation ${\sum }^{t_{r}}_{s=0}$ accumulates the impact of information up to and including time step *t*_*r*_. Furthermore, the impact of *misinformation* on user *i*, should be measured through all possible chances of being exposed to *misinformation*. That could be achieved by calculating the amount of malicious content from *n* adjacent users where user *i* is exposed to their content due to a direct following/ retweeting relationship. The overall average network impacts of both false and true content prior to and including the time stage *t*_*r*_ can be obtained by Eqs. 3, and 4, respectively, where |*U*| is the cardinality of the network users set.

3 $$  F^{t_{r}}= \frac{1}{|U|} \sum\limits^{|U|}_{i=1} F^{t_{r}}_{i} $$

4 $$  T^{t_{r}}= \frac{1}{|U|} \sum\limits^{|U|}_{i=1} T^{t_{r}}_{i} $$

To achieve actual mitigation during the spread of *misinformation*, a reasonable result would be by making $T^{t_{r}} \geq F^{t_{r}}$. That requires some intervention to change the counts which result in $T^{t_{r}}$. To apply such interference, we need to obtain the initial counts before modifying them. Therefore, a Hawkes process can be engaged to model the quantity of the generated content by each user at various simulation time stages.

#### Counting Generated Content

If we look at a point process on the non-negative real numbers line, where the latter is representing the time, the point process is a random process whose realizations *r* consist of the event times stages {*t*_0_, *t*_1_, ... *t*_*r*_} and they define the time by when an event has occurred. A point process on a specific time realization *t*_*s*_ can be redefined with an equivalent counting process. A counting process $N^{t_{s}}$ is a random function defined on a given time stage *t*_*s*_ ≥ 0, and takes the integer values {1, 2, 3, ...} as the number of events of the point process by the time stage *t*_*s*_. Hence, a random variable $N^{t_{s}}$ counts the number of events up to time *t*_*s*_ as the one below.

5 $$  N^{t_{s}} := \sum\limits_{i \geq t_{0}} \mathbbm{1}_{\{t_{s}\geq e_{i}\} } $$

Where *e*_*i*_ represents an event occurred by time *t*_*i*_ and $\mathbbm {1}_{\{.\}}$ is an indicator function that takes the value 1 when the condition is true, and takes the value 0 when it is false, making it a counting function with a jump of 1 within each time stage it counts for, while starting from the initial time stage *t*_0_ and finishing by the time stage *t*_*s*_.

The most straightforward class of point processes is the Poisson process. In Poisson processes, the random variables which represent the counts have an inter-arrival time, the rate of such arrivals per a time unit (stage) is denoted as *λ*, which refers to the intensity of the process. The latter is describing how likely and dense these counts or events to occur in a time sequence. However, in a Poisson process, the inter-arrival times are independent, in other words, the arrival of historical events do not influence the arrival of future events.

A well known self-exciting process was introduced by Hawkes (1971), the proposed model was based on a counting process where the intensity *λ* depends on all previously occurred events. In a Hawkes process, the arrival of an event shifts the conditional intensity function to an increase. Such a process determines its conditional intensity output based on two fundamental quantities, base intensity *μ*, and historical events arrival prior to a certain point in time $H^{t_{s}}$. With an analogy to *Twitter* and the problem of *misinformation*, the counts are the number of tweets, either true or dishonest ones. The base intensities can be viewed as the exogenous motivational factors which influenced a user to react, while the historical events can be viewed as the network endogenous factors, for instance, how the sub-network of user followees are acting on the network. In order to mimic *Twitter* dynamics as an environment for our mitigation method, we consider Multivariate Hawkes Processes (MHP) by defining U-dimensional point processes $N^{t_{s}}_{U}$, where *U* is the network users set, which emphasizes the self-excitation between events on social media (Zhou et al. 2013). $N^{t_{s}}$ can be interpreted as $F^{t_{s}}$ or $T^{t_{s}}$ as described in Eqs. 1 and 2, while |*U*| is the number of individual users a single Hawkes process is associated with, and *t*_*s*_ is the specific time realization or stage. The best way to describe a Hawkes process, is by its conditional intensity function as per (6).

6 $$ \lambda_{i}(t_{s}|H^{t_{s}})= \mu_{i} + \sum\limits_{t_{s^{\prime}}<t_{s}} g(t_{s}-t_{s^{\prime}}) $$

Where *g* is some kernel function over the history associated with user *i* from the time stage $t_{s^{\prime }}$ prior to time *t*_*s*_. *g* is concerned with the history of some influence *A*_*i*._. We used an exponential decay kernel function *g* = *A*_*i*._*e*^−*wt*^ as practiced in Farajtabar et al. (2017), where *w* is the decay factor which represents the rate for how the influence is reduced over time. For all *U*, the base intensity vector *μ*, and the influence matrix *A* can be estimated using maximum likelihood as presented in Ozaki (1979). To simulate *Twitter* dynamics for our mitigation task, we can rewrite (6) as the one below.

7 $$  \lambda_{i}(t_{s}|H^{t_{s}})= \mu_{i} + {\int}^{t_{s}}_{0} g(t_{s}-t_{s^{\prime}}) dN(t_{s^{\prime}}) $$

Where $N(t_{s^{\prime }})$ is the integration variable and the count of the historical generated content that influences user *i* and determined by the Hawkes process. In turn, the conditional intensity $\lambda _{i}(t_{s}|H^{t_{s}})$, tells how likely user *i* would act and generate content herself, by the time *t*_*s*_. Since $N(t_{s^{\prime }})$ is interpreted as $F(t_{s^{\prime }})$ and $T(t_{s^{\prime }})$ and can be calculated from Eqs. 1 and 2, it is important to highlight that the influence matrix *A* is considered explicit influence (following/ retweeting) when measuring content impacts on users after the simulation. On the other hand, *A* is considered implicit or hidden temporal influence when calculating the conditional intensity function, since the latter is a result of the estimated Hawkes parameters before the simulation, and indicating the independence from the network explicit structure. That is, we estimate the simulation parameters to obtain reasonable and inferred simulated network dynamics from the hidden temporal influence, then, we measure content impacts based on the explicit relationships on the simulated network dynamics.

#### Limited Budget Mitigation

To observe the process of the intervention-based mitigation, we followed a social network reshaping approach as employed in previous work (Farajtabar et al. 2017; Goindani and Neville 2019; Valera et al. 2015). To achieve such a resolution on the network, we are interested in the base intensity *μ*, since it defines any external motivation on the users. Hence, we are interested in adjusting the value of the base intensity by increasing it. However, there are two main challenges in this method, not all users would respond to an exogenous motivation, and not all of them are capable of boosting the activity of the network. Besides, some users would be spreading *misinformation* on purpose and they will not respond to an opposite campaign. Moreover, the time spent for incentivizing users is limited due to the crisis time criticality. Therefore, the modification of *μ* is bounded by a small amount of incentivization that should be allocated wisely among users to reach the optimum mitigation results.

Let us denote *C* as the optimization constraint, which represents the limited budget of incentivization. The optimization objective is to minimize the difference between *misinformation* and valid information impacts on the network by incentivizing the true content simulation-base intensities of users with respect to *C*. We define the optimization problem as a stochastic knapsack problem (Ross and Tsang 1989), where the selection of some users at a specific time stage is aimed in order to maximize the mitigation performance. The stochastic knapsack solution is bounded by the maximum allowed amount the knapsack can afford, in our case, this is referred to as *C*.

The purpose of the knapsack optimization is to fill a knapsack with materials amounts *X* = {*x*_*i*_,...,*x*_*n*_} such that they maximize some value $\mathcal {F}(X)$ but, at the same time, staying within the limited capacity of the knapsack (${\sum }^{n}_{i=1}x_{i} = C$). With an analogy to our problem, we can define the below minimization objective and constraint functions.

8 $$ \begin{array}{@{}rcl@{}} \min \mathcal{F}(X)&=&\sum\limits^{|U|}_{i=1} \mathcal{F}(x_{i}), \text{where } \ \mathcal{F}(x_{i})\\&=& \frac{1}{|U|} \sum\limits^{|U|}_{i=1} F^{t_{r}}_{i} - \frac{1}{|U|} \sum\limits^{|U|}_{i=1} T^{t_{r}}_{i}, \end{array} $$

9 $$  \text{subject }\ \text{to } \ \sum\limits^{|U|}_{i=1}x_{i} = C, \text{where } \ x_{i} > 0 : \forall u_{i} \in U $$

Where *x*_*i*_ is the incentivization amount for the user *i* and both $F^{t_{r}}_{i}$ and $T^{t_{r}}_{i}$ are random variables generated from a Hawkes count process *N*(*t*_*r*_) prior to realization *r* at time. Where $F^{t_{r}}_{i}$ is calculated through the simulation (7), and $T^{t_{r}}_{i}$ is calculated through the simulation (10), with replacing *N* by *F* and *T* in both equations, respectively. Therefore, the optimization problem is stochastic with regard to the objective function. Hence, and by finding the optimum incentivization amount *x*, the intervention can be applied by employing another Hawkes process for each user as the one below.

10 $$  \lambda_{i}(t_{s}|H^{t_{s}})= x_{i} + \mu_{i} + {\int}^{t_{s}}_{0} g(t_{s}-t_{s^{\prime}}) dN(t_{s^{\prime}}) $$

Where $N(t_{s^{\prime }})$ represents the count of true information events in the Hawkes model prior to the specified time stage *t*_*s*_, giving that $t_{s^{\prime }} < t_{s}$, and *t*_*s*_ ≤ *t*_*r*_ when r realizations (time stages) are the time intervals of the whole process.

### Paper Contribution and Limitation

It is essential to highlight that our approach is different from traditional approaches for finding graph centrality measures or most influential users on a social network (Rios et al. 2019; Fang et al. 2014). That is because our method would be under-performing if applied to a network where most influential users are spreading fake news. On the other hand, our purpose is to learn normal users who can be effective at a specific moment and independently from the graph structure and network centrality measures. Such independence is a crucial advantage of our approach, since it allows for further exploration and analysis of the temporal hidden influence structure on social networks. Besides, the timing driven feature is fundamental to crisis mitigation applications.

This paper introduces an adaptative learning method to achieve stochastic optimization over a social network. The optimization task is constrained and stochastic regarding its objective function. We applied our experiments on *Twitter* data, and evaluated our model on two publicly available real-world datasets that were used in previous work. Namely, *Twitter15* and *Twitter16* datasets (Liu et al. 2015; Ma et al. 2016, 2017). Moreover, we introduce a new *Twitter* dataset for the *COVID-19* pandemic. The latter represents a different situation that would demonstrate the flexibility of our solution. Results showed that with our light-weight computation method, we were able to find at least a local minimum that serves the required mitigation aims.

In our solution, we used a learning automaton (LA) (Thathachar and Sastry 2002) as an adaptative learning technique. An LA is a stochastic model, operating in the framework of reinforcement learning. The LA has been found to be a robust method for solving many complex and real-world problems where randomness is a primary characteristic of the problem. We built a network of LA, while each is assigned to a user on the social network. The individual LA should learn if the associated user is a good candidate for the mitigation campaign or not. Additionally, each LA determines an amount from a limited budget. The amount reflects how much we can spend from a limited budget. The latter can be viewed as an optimization constraint and the capacity of how likely we would depend on each of the suggested candidate(s), who would be part of an intervention process.

Our LA-based method differs from previous *misinformation* mitigation with reinforcement learning approaches (Farajtabar et al. 2017; Goindani and Neville 2019). The latter had a three dimension state per a user at a time, where user amounts of true and false events were observed with number of ”like” responses received. Then, the task was to learn a mitigation policy over the constructed state space. On the other hand, we redefine the task as a natural optimization problem, and we reconsider the problem of state space from being multidimensional to a single dimension, considering an overall network objective function with one single variable instead of calculating a multidimensional function across all users. Therefore, our objective function is only calculating one single value per a user, that drastically reduced the solution state space required for convergence, since the number of users on the network becomes the size of the state space. That means linear state space increase instead of exponential in case of scaling up the solution. We present our empirical results that show how that led to a faster and more reliable resolution without a notable loss in accuracy, where differences between users exposures to fake and true news have no skewed distribution.

We propose a novel exercise of the Learning Automata (LA) in the domain of social media *misinformation* mitigation. To the best of our knowledge, LA-based approaches were not employed in that area, and this paper is the first to conduct an LA study on online *misinformation* mitigation tasks. We approach that by evaluating three primary learning schemes for the LA. Moreover, and compare to similar mitigation approaches (Farajtabar et al. 2017; Goindani and Neville 2019). However, as a limitation in our work, we do not consider the political bias of users, compared to what has been done in Goindani and Neville (2019), in addition, we focus on non-skewed data points distribution scenarios where an average value could be the performance measure. Hence, political bias and skewed data scenarios are left for future improvements.

We evaluate our method and our implementation of the Hawkes processes-based simulation on two baselines datasets (*Twitter15*, *Twitter16*) after applying some post-processing on the original data. Furthermore, we introduce a new dataset (*Twitter-COVID19*) which was collected and annotated by us, and represents a different definition from the traditional fake news cases. The new dataset demonstrates the applicability and flexibility of our approach in different situations, such as the infodemic of *COVID-19*. In such a scenario, a mitigation task would be targeting the reduction of some propagated content effects, for instance, the irrational statements about an already-found cure or any incorrect crisis relief content that might motivate people to be less careful. Our experiments show promising results on all the evaluated datasets.

### Paper Organization

This paper is organized as follows. Section 2 introduces a literature review, where some of the previous adaptation of Hawkes processes are mentioned. Furthermore, other intervention-based mitigation approaches are briefly explained. The applied method and datasets are explained in Section 3, a statistical comparison between the datasets is demonstrated as well. Section 4 shows our empirical results and performance metrics. A discussion with comparable results to other mitigation methods is demonstrated in Section 5. Eventually, we conclude the paper and highlight possible future work in Section 6.

## Related Work

The definition of fake news or *misinformation* has evolved through time, not only due to the increased complexity of such social problem but also because of how recent technological efforts have progressed. For a long time, the spread of fake news on social media has been considered as the intentional dissemination of false information in news articles (Allcott and Gentzkow 2017). However, other research work started to give an attention to the broader scope of the problem (Sharma et al. 2019; Shu et al. 2017). For instance, rumor detection (Zhang et al. 2015), malicious accounts classification (Zannettou et al. 2019; Shao et al. 2018), and the causal aspects of *misinformation* (Abouzeid et al. 2019). However, and to the best of our knowledge, it has been an obstacle to effectively solve the problem in real-time or without data selection-bias concerns. Moreover, ethical questions are being asked (Wasike 2013) since fake news detection solutions are judgemental by nature. Therefore, the need for safer and online strategies that would lead to more generic and authentic resolutions are critically desirable.

As a motivation for more online resolutions to social media *misinformation*, intervention-based mitigation strategies have been practiced in the literature. Reshaping users activities by applying an interference strategy was introduced in Farajtabar et al. (2014). In addition, dynamic programming was employed to optimally distribute incentivization resources among users in different time stages (Farajtabar et al. 2016). In such previous work, objective functions were designed using expected values of exposure counts of the user content, generated from a Hawkes process. The latter has been applied as a simulation for the social media information diffusion in many recent applications as well (Zadeh and Sharda 2014; Kobayashi and Lambiotte 2016). Multivariate Hawkes Processes (MHP) have proven efficiency and robustness in social media analysis and more specifically in the domain of *misinformation*.

Since recent advances in *misinformation* mitigation approaches have achieved an online and interactive (simulation-based) resolutions (Farajtabar et al. 2017; Goindani and Neville 2019), future work would focus on improving and wider applications rather than a narrow definition of *misinformation* or limited datasets that were examined in previous work (Liu et al. 2015; Ma et al. 2016, 2017). For example, the conducted intervention-based mitigation could be used for polarization, hate speech, and infodemics mitigation resolutions. The latter is one of our interests at this study.

The use of reinforcement learning for *misinformation* mitigation on social media has revealed a promising future for how such a problem could be tackled. However, to model a large state space as practiced in Farajtabar et al. (2017) and to evaluate an optimum policy through that, is still a big concern, especially for a mitigation task that needs to be achieved timelessly. Moreover, user incentivization should be applied according to the problem context, which causes a loss of generality in some cases. For example, in a political scenario, a mitigation objective function would consider the political bias of users (Goindani and Neville 2019) before preference them as suitable candidates, since users with an ideological bias would not respond to the incentivization (Allcott and Gentzkow 2017). The conducted study in this paper aims to provide a light-weight computation framework that could be applied to different mitigation contexts without a total re-engineering effort. However, we consider this paper as the first step for our proposed system structure by evaluating a network of learning automata (LA) where a light-weight LA is the core of our framework. Therefore, the political bias of users is not investigated so far.

An LA is an adaptative learning method which can be viewed as a stochastic model operating in the framework of reinforcement learning. An LA has been found to be a robust method for solving many complex and real-world problems where randomness is a primary characteristic of the problem. Previous applications of LA have been introduced for social network analysis problems. For instance, a stochastic learning-based weak estimator for learning and tracking a user’s time-varying interest was practiced for social media-based recommendation systems (Oommen et al. 2012). An LA-based framework was also employed for online service selection in a stochastic environment where the latter has unfair service reviews (Yazidi et al. 2012). A stochastic constraint optimization problem such as the one approached in this paper could be defined as a stochastic knapsack problem, where LA has been tasked for by employing the LA Knapsack Game (LAKG) (Granmo et al. 2007).

LAKG is a game between *n* finite automata that interact with a scheduler and a stochastic environment. The stochastic environment consists of a set of stochastic material unit volume value functions. If an amount of a certain material is suggested to the environment and favored, the associated value function takes the value 1 with probability *p* and the value 0 with probability 1 − *p*. Besides, the environment provides a signal *ϕ*, which indicates whether the knapsack is full or not, which also tells if the optimization constraint was reached or not. On the other hand, the scheduler takes material amounts as its input. The purpose of the scheduler is to perform the access to the stochastic environment, sequentially. Besides, it makes sure that the unit volume value functions are accessed with frequencies proportional to all materials amounts. Such a problem description is similar to an incentivization across *n* users, where the incentivization budget is limited. Therefore, we consider a learning scheme similar to Granmo et al. (2007). However, in that knapsack problem, *n* materials can be evaluated through the whole problem space. Still, on a large social network, it would be impossible to assess a value function across the entire problem space. Therefore, we adapted a different structure to face such an obstacle. Moreover, due to our problem specification, we distinguished between the learned and the evaluated material amount for the value function.

## Methodology

### Learning Automata

A Learning Automaton (LA) is a model of intelligent computation where learning is accomplished by exploring and its consequences, in an iterative and reinforcement manner. The objective is to decide the optimal action to select between all the possible actions. An LA learns by interacting with an environment. The environment sends a regular feedback for when the LA explores a particular action. The LA estimates its preferred action selection in future explorations with regard to the environment recent response.

**Fig. 1:**
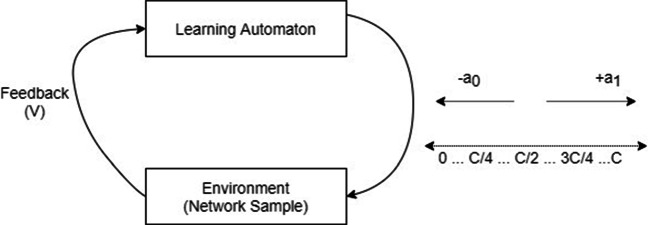
LA interaction process

Social media platforms and mainly in an emergency setting, are considered as random mediums where uncertainty and outliers exist. Such uncertainty and randomness motivated this paper to practice an LA-based optimization framework as a network of LA, while each LA is assigned to a user on the social network to learn about its authenticity probability of being part of a *misinformation* mitigation strategy. Figure 1 shows how an individual LA works by interacting with an environment. Each LA task is to learn about the best action between two possible moves. That is, each LA has two possible moves (*α*_0_, *α*_1_) over an associated random walk line, representing moving in the direction of assigning less incentivization and the direction of assigning more incentivization from a mitigation budget *C*, respectively. For a given *LA*_*i*_ and its chosen action *α*_1_ and incentivization amount *x*_*i*_ at an epoch i, the environment sends the feedback $V^{\mathrm {i}}_{i}$ according to Eq. 11.

11 $$  V^{\mathrm{i}}_{i}= \begin{cases} 1,& \text{if } \ \mathcal{F}^{\mathrm{i}}(x_{i}) < \mathcal{F}^{\mathrm{i}}(x_{j})\\ 0,    & \text{otherwise} \end{cases} \text{, where}\ i \neq j $$

Where $\mathcal {F}^{\mathrm {i}}(x_{i}), \mathcal {F}^{\mathrm {i}}(x_{j})$ are some investigated objective functions at epoch i for *LA*_*i*_ and *LA*_*j*_ at the epoch iterations *i* and *j*, respectively. An objective function calculation is done as per our mitigation objective function definition in Eq. 8. However, with adapting such calculation in our framework, and for a faster computation, we practically reduce the size of the network by randomly sampling over a subset of users *U*^−^, while |*U*^−^| is selected according to the minimum subset size which does not sacrifice the accuracy of the results, since larger subsets might improve the process of user evaluation but will slow the computation. For that, we conducted a grid search to estimate the best value of |*U*^−^|. A detailed grid search result and the final selected hyper parameters values are demonstrated in section 4, with respect to the convergence rate and the mitigation performance metric. Considering a network sample |*U*^−^| instead of the whole network size |*U*|, an objective function at a given epoch i can be redefined as the one below.

12 $$  \mathcal{F}^{\mathrm{i}}(x_{i}) = \frac{1}{|U^{-}|} \sum\limits^{|U^{-}|}_{i=1} F^{\mathrm{i}}_{i} - \frac{1}{|U^{-}|} \sum\limits^{|U^{-}|}_{i=1} T^{\mathrm{i}}_{i} $$

For each an epoch i inside a specific time stage *t*_*s*_, all LA are sequentially visited one time. Therefore, the function $\mathcal {F}^{\mathrm {i}}(x_{j})$ is also calculated and compared with $\mathcal {F}^{\mathrm {i}}(x_{i})$, to evaluate how two mitigation functions are different in terms of a minimum value, since the target is to minimize the overall network difference ($F^{t_{s}} - T^{t_{s}}$) while learning the optimum subset of users *U*^∗^(*t*_*s*_).

The environment feedback *V* is stochastic since the mitigation sub-functions are a result of a stochastic process, namely, a multivariate Hawkes process (MHP). Therefore, the challenge of our stochastic optimization framework is to learn how to minimize $\mathcal {F}(X)$ under the constraint *C* as discussed in Eqs. 8 and 9. During the learning process, the value *X* is determined by a learning rate *η*, as a constant per all users, time stages, and epochs. For instance, while *i*≠*j*, *x*_*i*_ = *x*_*j*_ = *η*, since evaluating different users should be fairly applied by assigning the same incentivization amount. The hyper parameter *η* can be estimated through our grid search as well. On the other hand, and per each user, the determined *X* for the mitigation will be the final converged state of each *LA*, since that indicates an amount determined through the LA interaction with the environment. Hence, the final converged network incentivization values is a random vector which represents the converged states of the individual LA over a random walk line for each. Section 4 gives more details about how different the learning rate *η* could be on each dataset and what are the factors that dictates its values.

**Fig. 2:**
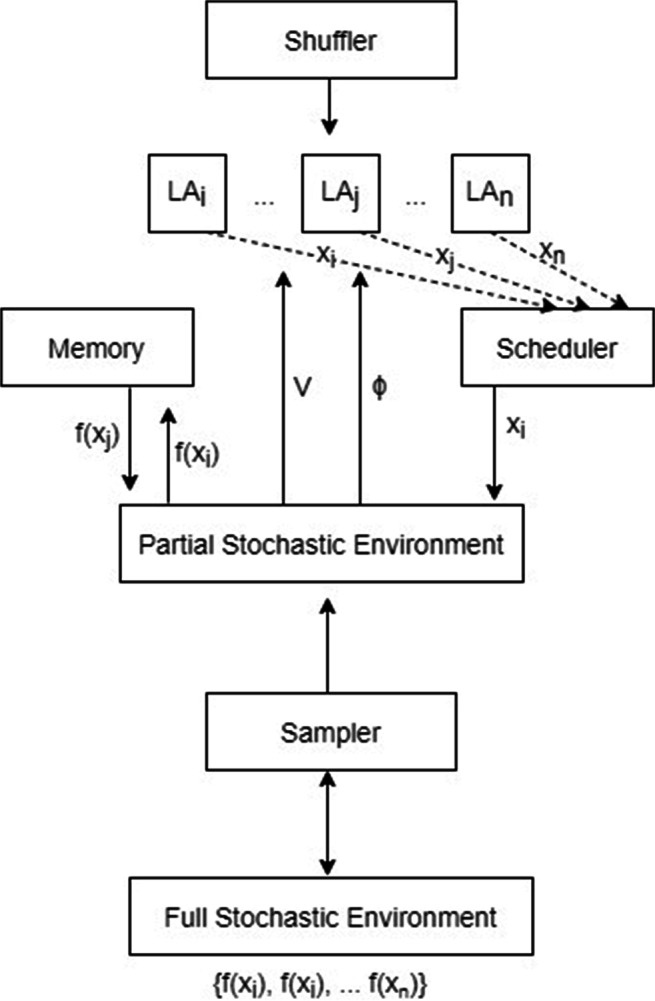
Mitigation framework structure

We designed the network with a uniform learning scheme for the individual LA. Furthermore, we followed a simple random walk as in Granmo et al. (2007) to represent the LA state transitions. However, our individual LA are different in the way they interact with the environment and in their structure as well. In our case, the environment is partially observed, we refer to that by network sample size |*U*^−^|. We have evaluated three main learning schemes for our framework, we refer to them as *random walk reward-penalty in actionRW*_*RP*_, *random walk reward in action* *RW*_*RI*_, and *random walk penalty in action* *RW*_*PI*_. Figure 2 demonstrates the components of our framework.

As one of the components in our framework, a shuffler, which is triggered every an epoch after all LA are visited, to ensure the comparison pair $\mathcal {F}^{\mathrm {i}} (x_{i}), \mathcal {F}^{\mathrm {i}}(x_{j})$ will be different each time. The sampler component selects a subset of the overall network with size |*U*^−^|, determined by a hyper-parameter in our configuration. The scheduler component maintains sequential and equal visits to each LA to guarantee equal potential state transitions and actions probabilities update behaviour. The memory component at each computation time step inside an epoch, helps the sampled partial environment to send its feedback according to the evaluation of the functions $\mathcal {F}^{\mathrm {i}}(x_{i}), \mathcal {F}^{\mathrm {i}} (x_{j})$.

For our optimization framework to work efficiently, each LA should have two updating rules, the first is the LA action probability update rule, the second is the state transition mechanism, which eventually decides the amount of incentivization that will be assigned for each user. Therefore, the incentivization amounts are considered shifts (left/ right) in a line where a random walk is exercised. The line represents the LA state space. The shifts are probabilistic and subject to the LA action probabilities and the signal *ϕ*. The latter tells the LA if the budget constraint *C* has been met or not yet. Each LA state space has its own boundaries from 0 to *C*, indicating a minimum and a maximum allowed state values, respectively. Hence, the actions (moves) probabilities are updated according to the environment feedback *V* at each individual LA visit (epoch). Equation 13 describes the action probability updating rule. The higher the probability of moving to the right ($\alpha _{i_{1}}$), the more likely the user *i* is a good candidate for the mitigation.

13 $$ \begin{array}{@{}rcl@{}} \mathcal{P}^{\mathrm{i}}(a_{i_{1}})&=& \frac{W^{\mathrm{i}}_{i_{1}}}{Z^{\mathrm{i}}_{i_{1}}} \text{, and } \ \mathcal{P}^{\mathrm{i}}_{i}(a_{i_{0}})= 1 - \mathcal{P}^{\mathrm{i}}_{i}(a_{i_{1}}) \text{, where }\\ W^{\mathrm{i}}_{i_{1}}&=& \sum\limits^{\mathrm{i}}_{e= 1, e\leq \mathrm{i}} {V^{e}_{i}} \end{array} $$

Where $W^{\mathrm {i}}_{i_{1}}$ and $Z^{\mathrm {i}}_{i_{1}}$ are counters for how many times the action $a_{i_{1}}$ was rewarded and selected starting from first epoch *e* and till the current epoch i, respectively. The *RW*_*RP*_ learning scheme is in action when *LA*_*i*_ moves are either rewarded or penalized. Hence, the actions probabilities are updated all the times when *LA*_*i*_ interacts with the environment. Algorithm 1 (see A A) gives the complete details of how *RW*_*RP*_ works and how the state transition is applied.

**Figure:**
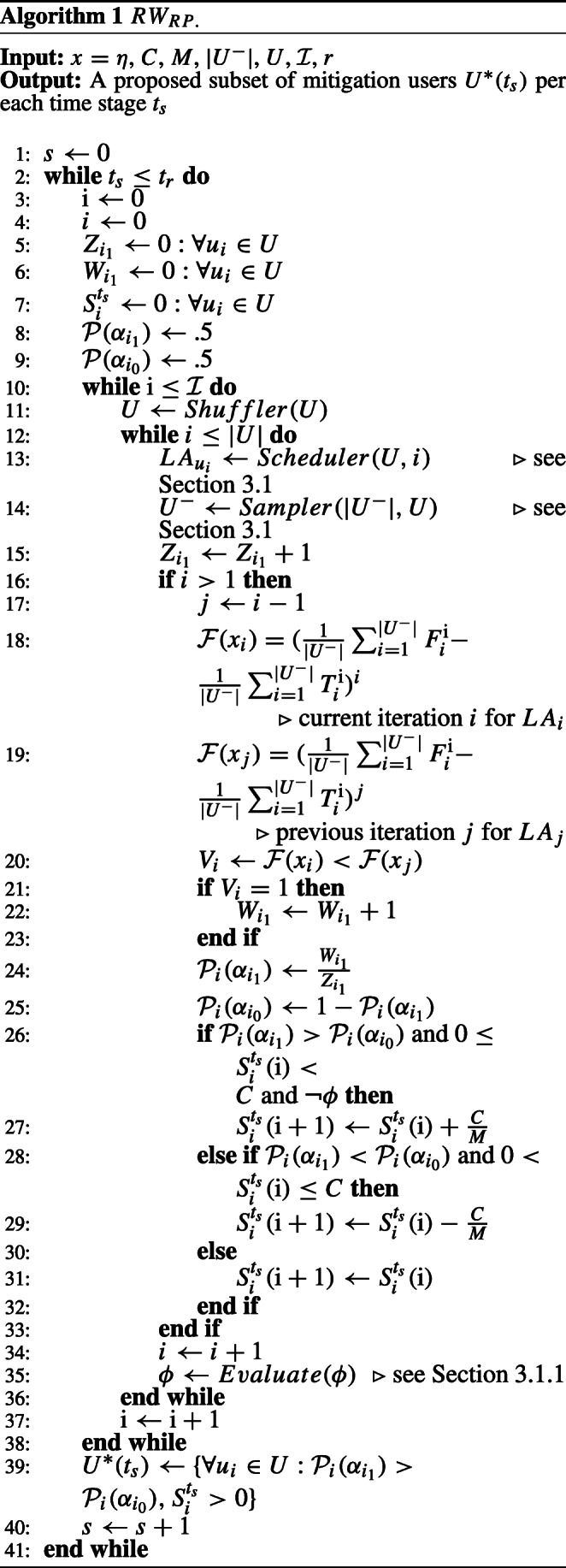

Differently, the *RW*_*RI*_ learning scheme is in action when *LA*_*i*_ moves are only rewarded. Hence, the actions probabilities are updated only when $V^{\mathrm {i}}_{i} = 1$, according to the recent interaction feedback of *LA*_*i*_ with the environment. Algorithm 2 (see A A) demonstrates how *RW*_*RI*_ works and its state transition.

**Figure:**
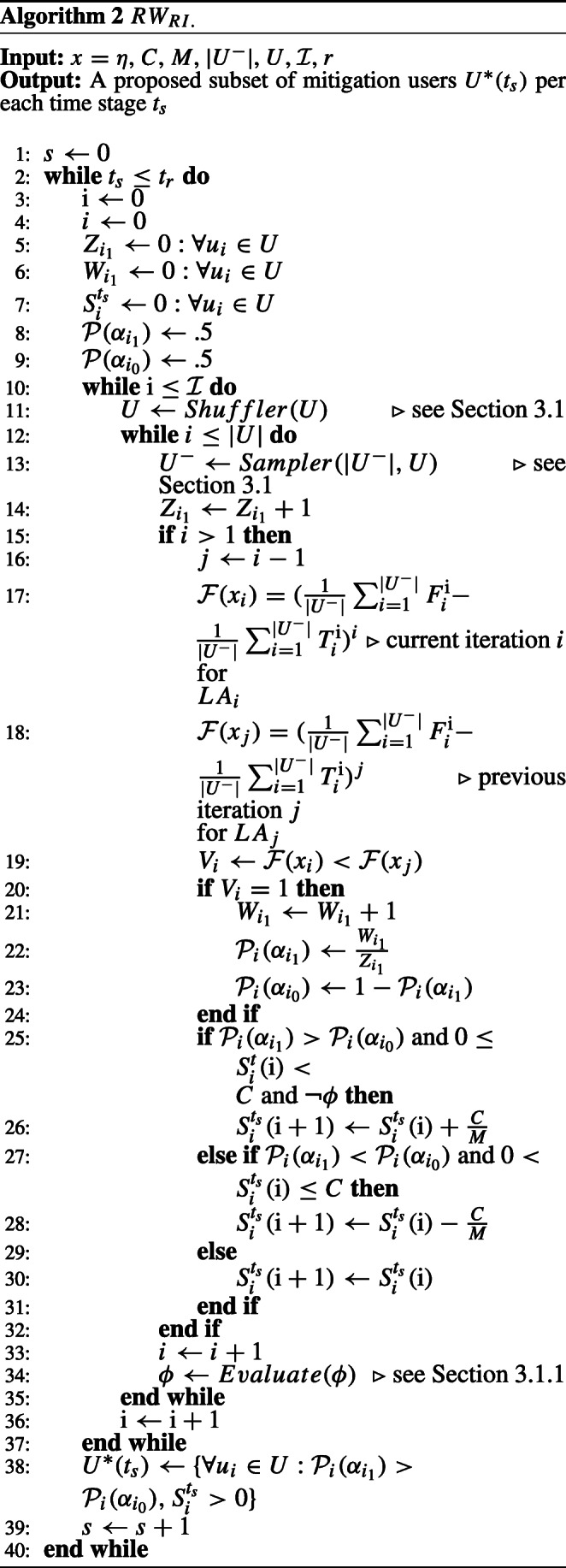

The *RW*_*PI*_ learning scheme works similar to *RW*_*RI*_ except the former is in action when *LA*_*i*_ moves are only penalized. Which causes the actions probabilities to be updated only when $V^{\mathrm {i}}_{i} = 0$. Algorithm 3 (see A A) gives the complete details of the state transition and how *RW*_*PI*_ works. For any LA, since our purpose is to evaluate for the incentivization, the action *α*_1_ is always performed for all our learning schemes, and $\mathcal {P}(\alpha _{0})$ is only updated as per (13).

**Figure:**
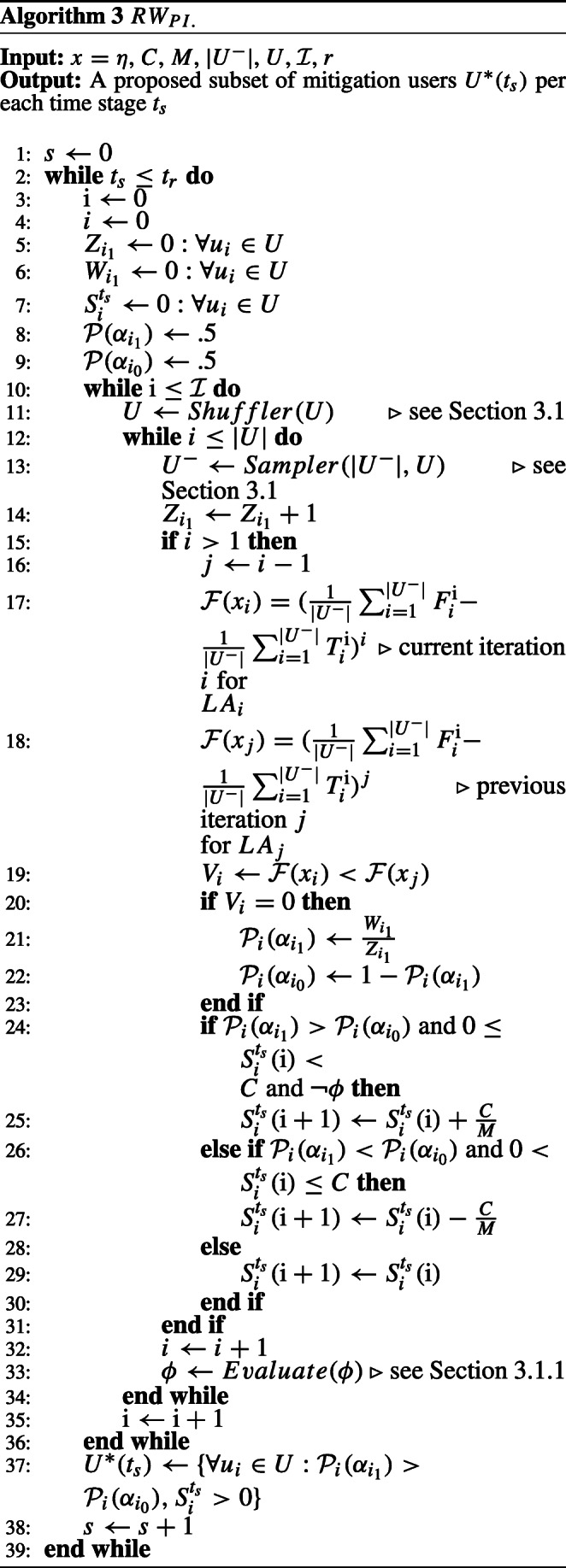

#### Random Walk Learning

As a result of learning the incentivization amounts per users, by the end of all computation steps of $\mathcal {I}$ epochs on each time realization *t*_*s*_ of the Hawkes process, each LA suggests if its associated user would be part of a proposed subset *U*^∗^(*t*_*s*_) of mitigation candidate(s). Where $\forall u_{i} \in U: u_{i} \in U^{*}(t_{s}), x_{i}=S_{i}(t_{s}) \text {, if } \ \mathcal {P}(\alpha _{i_{1}}) > \mathcal {P}(\alpha _{i_{0}})$ by the end of the computation, and *S*_*i*_(*t*_*s*_) is the final converged LA state value of user *i* at the time realization *t*_*s*_. Therefore, *S*_*i*_(*t*_*s*_) will be the final decided assigned value to the variable *x*_*i*_ for the intervention process as demonstrated in Eq. 10.

In the core of our proposed framework, there is a decentralized LA learning model, which learns such final incentivization amount for a user. The learning model learns by performing stochastic moves (actions) over a state space (possible incentivization amounts). The stochastic moves $\mathcal {P}(\alpha _{i_{1}}), \mathcal {P}(\alpha _{i_{0}})$ are determined as per (13), which is dependant on the environment feedback which is measured according to Eqs. 11 and 12. Despite how the random walk moves probabilities are being updated through the different learning schemes (*RW*_*RP*_, *RW*_*RI*_, *RW*_*PI*_), at a specific MHP time stage, and an epoch i, the user *i* associated LA model learns by conducting random walk moves as the below formal description.

$$ \begin{array}{@{}rcl@{}} S^{t_{s}}_{i}(\mathrm{i}+1) := S^{t_{s}}_{i}(\mathrm{i}) + \frac{C}{M}, &&\text{if } \mathcal{\ P}(\alpha_{i_{1}}) > \mathcal{P}(\alpha_{i_{0}}) \text{ and } \\&& 0 \leq S^{t_{s}}_{i}(\mathrm{i}) < C \\&&\text{and } \neg \phi \\ S^{t_{s}}_{i}(\mathrm{i}+1) := S^{t_{s}}_{i}(\mathrm{i}) - \frac{C}{M}, &&\text{if \ } \mathcal{P}(\alpha_{i_{1}}) < \mathcal{P}(\alpha_{i_{0}}) \text{ and } \\&& 0 < S^{t_{s}}_{i}(\mathrm{i}) \leq C \\ S^{t_{s}}_{i}(\mathrm{i}+1) := S^{t_{s}}_{i}(\mathrm{i}), &&\text{otherwise,} \end{array} $$

$$ \begin{array}{@{}rcl@{}} \text{where } \phi = \begin{cases} true, &\text{if }\ \frac{C}{M} + {\sum}^{|U|}_{i=1} S^{t_{s}}_{i}(\mathrm{i}) > C \\ \\ false, &\text{otherwise.} \\ \end{cases} \end{array} $$

**Fig. 3:**
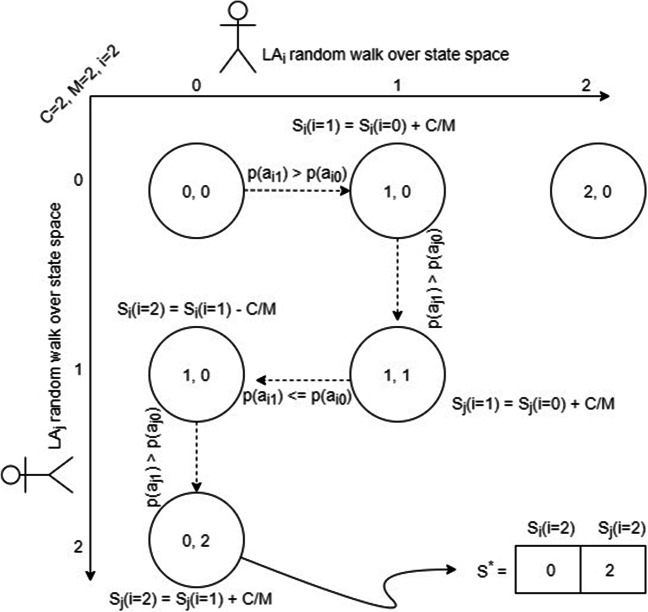
Toy Example of two LA-based joint random walk

Where *M* is the constant memory depth of each LA state space bounded by *C*, therefore $\frac {C}{M}$ describes the shift value resulted by the random walk. Since all users should be evaluated through their own random walk model, the above description is applied on all LA, sequentially through the scheduler component as indicated in Fig. 2. Moreover, Fig. 3 gives a toy example of how a joint random walk learning process from two sequential LA moves constructs the network converged incentivization vector *S*^∗^. Where the horizontal line indicates user *i* state space, and the vertical line indicates user *j* state space, given that the latter ended up being allocated all the incentivization budget (*C* = 2) after two epochs (i = 2) and 4 time steps. The example then can be generalized for as many number of users (LA). Eventually, it is important to highlight that at each time stage of the MHP, the individual LA moves probabilities $\mathcal {P}(\alpha _{i_{1}}), \mathcal {P}(\alpha _{i_{0}})$ are reset, to ensure learning new temporal influential users over different time stages, if exist.

#### Rate of Convergence

For an individual *LA*_*i*_, its convergence is defined according to the optimum incentivization value (random walk converged point) $S_{i}^{*}$. Hence, the whole LA network optimum random vector *S*^∗^ is considered the optimization minimizer vector. Therefore, the rate of convergence or the asymptotic error of the LA network can be defined as per (14), where i represents the current epoch (LA visit).

14 $$  \textit{error} := \frac{||S_{\mathrm{i}+1} - S^{*}||}{||S_{\mathrm{i}} - S^{*}||} $$

The above definition is then used for evaluating the network hyper parameters. Additionally, from our observation, at least for one of the applied LA learning schemes, we obtained a *Q-superlinear* rate of convergence for all datasets, where the network asymptotic error approached 0. A detailed explanation for the network rate of convergence using different hyper parameters values is given in Section 4.

### Datasets

#### Twitter15

The *Twitter15* dataset has initially been collected and created to debunk rumors on *Twitter* (Liu et al. 2015). The original dataset had both a political and a more general context. Two rumor tracking websites (*snopes.com and emergent.info*) were used to verify the trustworthiness of the content before categorizing the data into true, false, and unverified rumors. The results consisted of 94 true and 446 fake stories. Accordingly, all relevant and matched tweets were collected and labeled. After downloading and post-processing the original *Twitter15* dataset, we obtained 27,547 users, which contributed to 21,279 of true events (*tweets/ retweets*), and 6,268 of false ones. However, for our experiments and due to the current limitation in our computation power, we scaled down the size of the network to only 1,039 users, and 1,188 events, considering scaling that up in future experiments. Our scaled network also focused only on the American political context. Therefore, the final network was a result of extracting main tweets with relevant keywords and hashtags from the standard dataset. Hence, we only extracted main tweets that contain the keywords and hashtags as demonstrated in Table 1. The final network had approximately 94.02*%* of *misinformation*.

**Table 1:** Filtered *Twitter15/16* datasets tweets

Hashtag/ Keyword	(*Twitter15*) Number	(*Twitter16*) Number
	of main tweets	of main tweets
hillary	16	21
trump	27	37
obama	44	30
america	4	3
americans	12	7
american	19	3
democrat	5	5
republican	1	1
clinton	16	27
white house	2	39

For all datasets, we define the term *u*_*influencer*_ as the user with the highest node degree on the network, with regard to the number of edges which represent retweeting from her. In *Twitter15* dataset, the top *misinformation* influencer node motivated 301 users to spread false content. Also, 13 users were motivated to spread true news by retweeting valid content, since the top influencer user had generated some trustworthy content as well, that opens a judgemental question if such a user is spreading *misinformation* on purpose or not, and how much spread is enough to measure that in our study. We believe, such question is irrelevant to this study, however, we will involve these numbers when looking at the results and *U*^∗^(*t*_*s*_), since we aim to have an independence from the network high centrality node(s) when it is necessary.

#### Twitter16

The *Twitter16* dataset was collected initially for a recurrent neural network for rumor detection in social media (Ma et al. 2016). The data was evaluated by the online rumor debunking service (*snopes.com*), where 778 events were investigated during March-December 2015, and 64*%* of the data samples were actual rumors. Similar to *Twitter15*, the context of the events are broader than only political struggles. Hence, and after our post-processing, we ended up with 45,566 incidents and 44,114 users, which contributed to 38,686 non-rumors, and 6,880 rumors. However, and after scaling down and focusing only on political struggle related events, our final dataset version was 1,206 users and 1,362 cases, from which there were around 45.59*%* considered as*misinformation*. Table 1 shows the hashtags and keywords used for filtering the main tweets from the standard dataset.

The top *misinformation* influencer node incentivized 230 users to spread malicious content. However, the same user has also incentivized 52 users for retweeting correct information, which means she might not be spreading false content on purpose. Like in *Twitter15* dataset, that insight is useful when evaluating the performance of our method, since the latter should not be driven by such top nodes, especially when they are circulating fake content on purpose. Therefore, some relevant statistical measures would be useful to set a boundary for how we could accept the learned *U*^∗^(*t*_*s*_), in cases when *u*_*influencer*_ ∈ *U*^∗^(*t*_*s*_).

#### Twitter-COVID19

Our new proposed dataset *Twitter-COVID19* was collected during the 28th of March 2020 for the *COVID-19* infodemic on *Twitter*, the dataset had 1,164 users and 1,180 events, from which there were around 92.03*%*, manually labeled as *misinformation*. The dataset focused on the circulated irrational content about some found cures like ”silver liquids” and the ”anti-malaria” medication. The latter was reported as a cause of severe harmful side effects for people who tried it without consulting a health expert^2^. To show the flexibility of our approach, the mitigation resolution can be seen as applicable in any case where there are two opposite campaigns, and the task is to mitigate one in favor of the other. In our case, we consider a reduction of the effect of believing these false crisis reliefs, since there was no approved cure yet, by the time of collecting the dataset. That can be viewed as an exercise for risk reduction during an infodemic.

In *Twitter-Covid19* scenario, the top *misinformation* influencer node motivated 766 users to spread irrational content. The influencer node had no effect on spreading any other type of contents during the time window of collecting the data. Therefore, that is considered a perfect example to evaluate how our method would avoid such user before suggesting *U*^∗^(*t*_*s*_). Table 2 demonstrates some statistical differences between the three datasets.

**Table 2:** Datasets statistics

Dataset	*Twitter15*	*Twitter16*	*Twitter-COVID19*
Num of users	1,039	1,206	1,164
Number of events	1,188	1,362	1,180
*Misinformation*	94.02*%*	45.59*%*	92.03*%*
Network density	.001943	.001778	.001687
*Misinformation*	28.87*%*	19.07*%*	65.81*%*
by *u*_*influencer*_			

## Empirical Results

### *Twitter15*

**Fig. 4:**
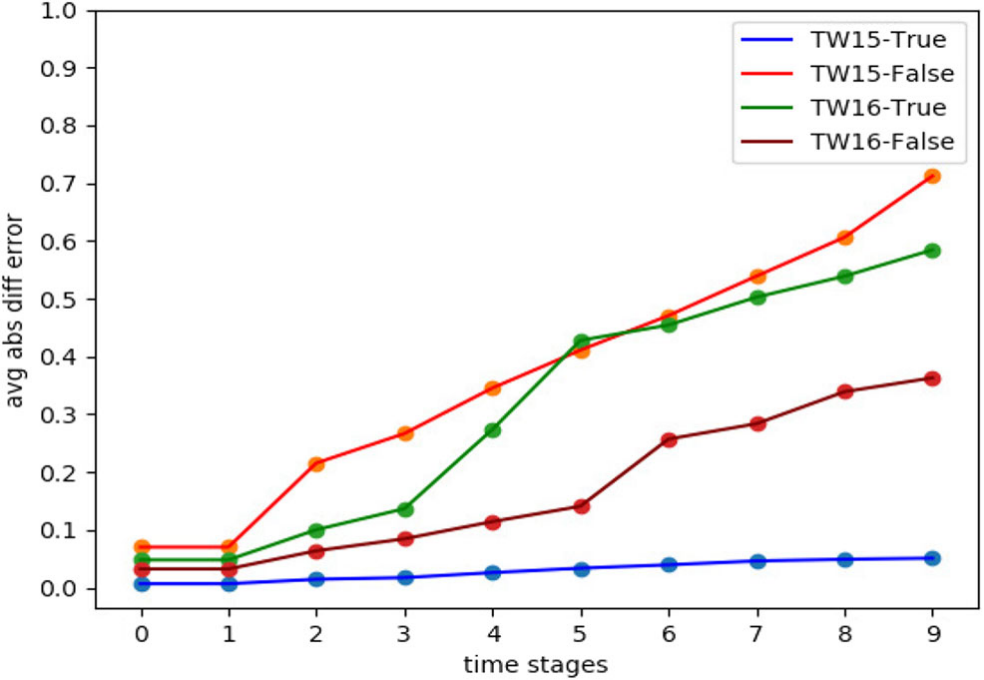
MHP simulation vs real data on scale of 1 less or more tweet per user difference on a time stage

Our first experiment was the *Twitter15* dataset, which had around 94.02*%* of *misinformation*. This is considered an important example for evaluating our algorithms on such high percentage. For the Hawkes processes, we set the decay factors *w* = .75 and *w* = 1 as in Goindani and Neville (2019) for false and true events, respectively. Besides, we set an hourly interval between time stages (Δ = 1 hour). From the dataset events timestamps, we used the first 10 hours for learning the Hawkes parameters *μ* and *A*, before simulating the next 30 hours. Therefore, we used the next 30 hours from the real data for testing, by comparing with events arrivals which were generated from the simulation. We obtained a relatively good simulation behaviour. Figure 4 indicates the average absolute difference error (Farajtabar et al. 2017) as the performance metric used for the simulation on both true and false events from *Twitter15* and *Twitter16* datasets. Equation 15 demonstrates how the average absolute difference error was calculated.

15 $$ \begin{array}{@{}rcl@{}} \mathcal{E}_{t_{s} + {\Delta}}&=& \frac{1}{|U|} \sum\limits^{|U|}_{i=1}|[N^{\mathcal{H}}_{i}(t_{s} + {\Delta}) - N^{\mathcal{H}}_{i}(t_{s})]\\&&-[N^{\mathcal{R}}_{i}(t_{s} + {\Delta}) - N^{\mathcal{R}}_{i}(t_{s})]| \end{array} $$

Where |*U*| is the number of users and $N^{{\mathscr{H}}}$, $N^{\mathcal {R}}$ represent the counts of the arrived events from Hawkes simulation and real data, respectively. The calculation is made between the time stages *t*_*s*_ + Δ and *t*_*s*_. It is important to highlight that we consider improving the simulation process in future, so that we could maintain a more stable error over time. However, we believe an error up to 1 is still a good indicator since for 1,000 users, that means, on average, there is only 1 event arrival difference per user and prior to a certain time stage. Additionally, we define a random count range for more convenient simulation results, the count range $N^{t}_{\mathcal {E}^{\pm }}$ interprets $\mathcal {E}$ as a possible discount in the number of generated events. For instance, if *N*^*t*^ = 50 from the simulation, we might be more confident to say $N^{t}_{\mathcal {E}^{\pm }}=[(1-\mathcal {E}) \cdot 50, 50]$. In case of $\mathcal {E} > 1$, it should be normalized between 0 and 1.

**Fig. 5:**
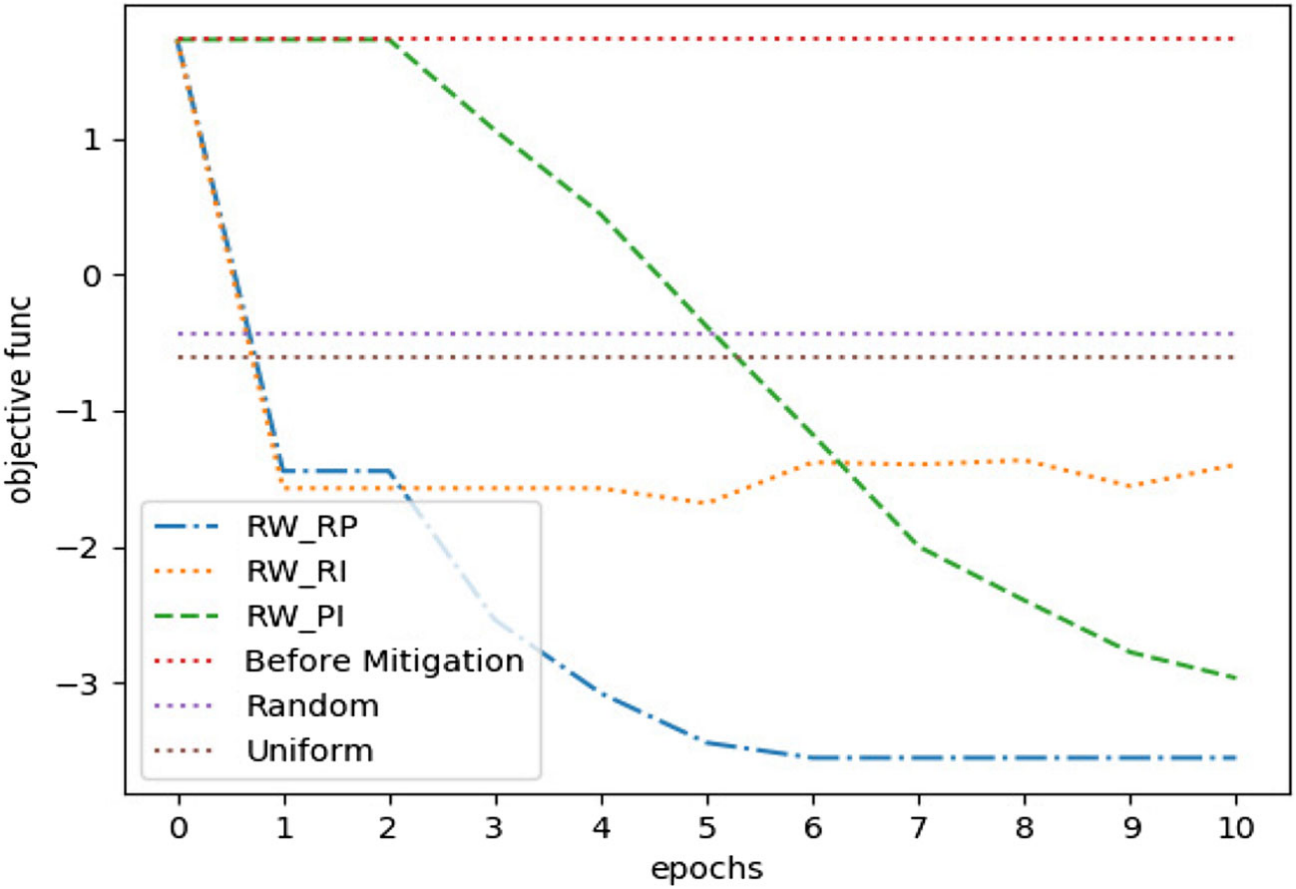
*Twitter15* mitigation performance on 1’st time stage, *C* = .05

**Fig. 6:**
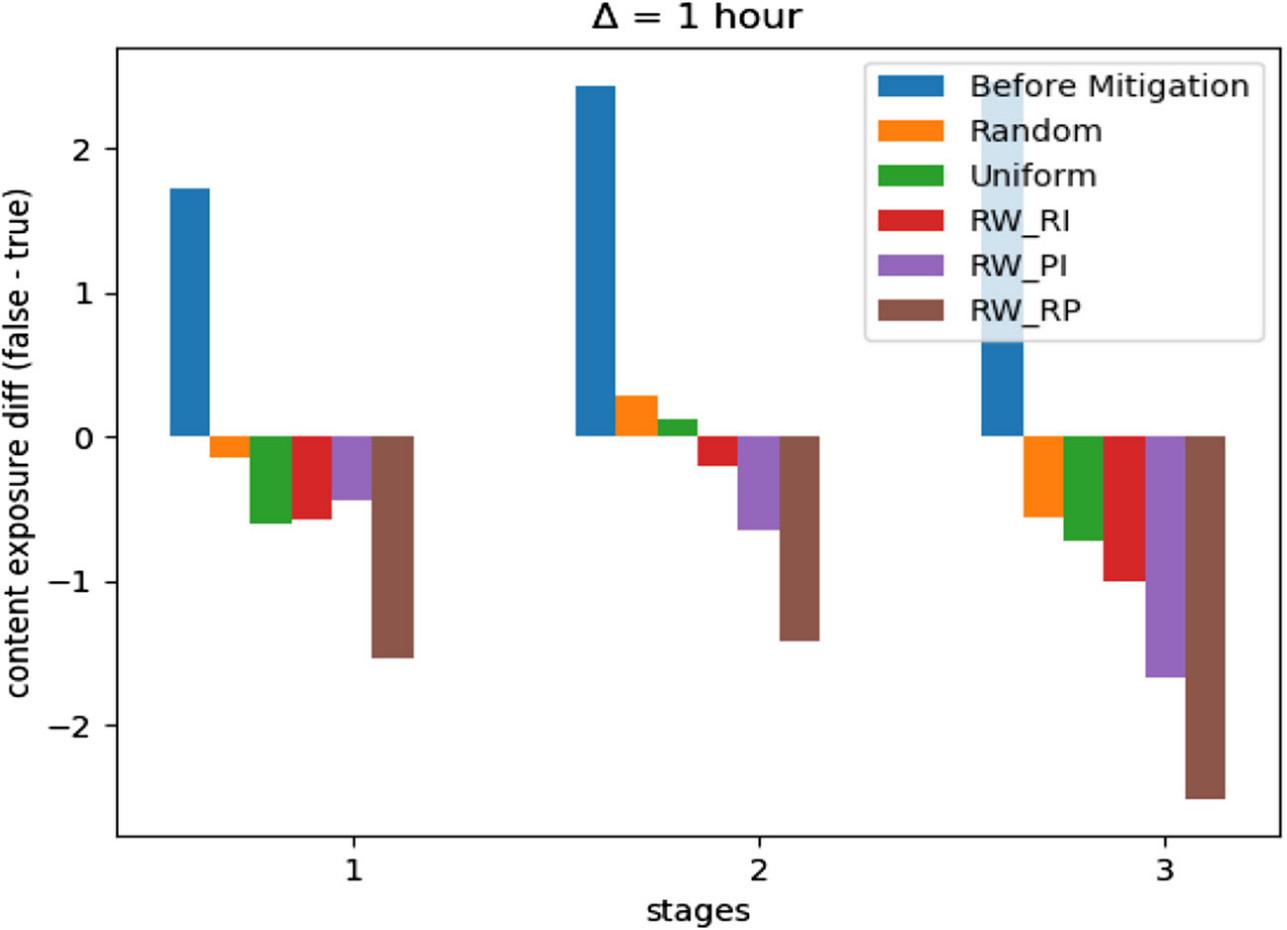
*Twitter15* mitigation performance on first three time stages, *C* = .05

For *Twitter15* dataset, Fig. 5 shows the optimization performance of the three suggested learning schemes with three other considerable performance baselines. The three baselines represent three different measures we sought to outperform, these are *misinformation* before mitigation, uniform distribution of incentivization budget, and random allocation of incentivization budget. Our optimization framework outperformed the three baselines with the three learning schemes with a budget *C* = .05. The latter is considered a limited budget according to its overall effect on the MHP. Moreover, we observed approximately similar performance between *RW*_*PI*_ and *RW*_*RP*_ on longer epochs, but *RW*_*RP*_ was the one with a remarkable early convergence. Eventually, Fig. 6 shows the performance for difference minimization between *misinformation* and true events after learning *U*^∗^ for the first three time stages (next three hours).

### *Twitter16*

The simulation driven from the *Twitter16* dataset can be evaluated as in Eq. 15. And as indicated in Fig. 4, the false events simulation seemed to be slightly enhanced compared to *Twitter15*.

Our version of the *Twitter16* dataset is considered as an interesting case, since it is a situation where *misinformation* is around 45.59*%* over the network, that is considered too low, relatively to both *Twitter15* and *Twitter-COVID19* datasets. However, it seemed that such scenario was more challenging. That is because when an extreme level of *misinformation* exists, it becomes straight forward to distinguish between nodes with temporal negative influence. On the other hand, when there is a balance between both campaigns in the network, it is more vague to distinguish between the authenticity of nodes, since all nodes might be contributing to both *misinformation* and true information diffusion. Moreover, it becomes interesting to see how our method was independent from the network central node(s) in such cases. The latter perspective is fundamental, as it would indicate how flexible and intelligent our method is. For instance, sometimes in a scenario like *Twitter15* and *Twitter16*, a top influencer node might be spreading false content, but still, it is circulating true content.

For the Hawkes processes, we set the decay factors *w* = .6 and *w* = 1 as in Goindani and Neville (2019) for false and true events, respectively. Besides, we set an hourly interval between time stages (Δ = 1 hour). We used the same duration as in *Twitter15* for both learning Hawkes parameters and testing.

**Fig. 7:**
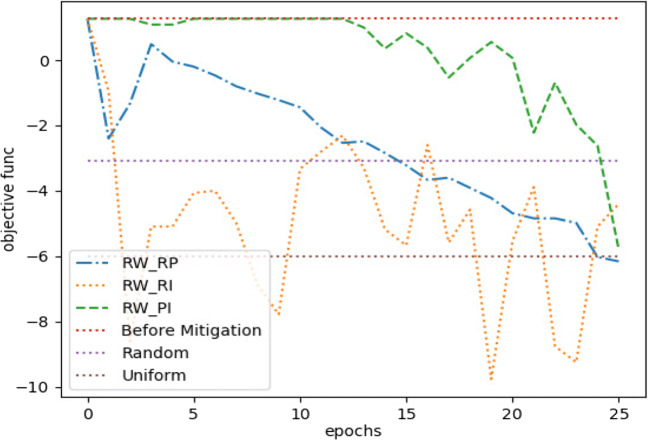
*Twitter16* mitigation performance on 1’st time stage, *C* = .05

**Fig. 8:**
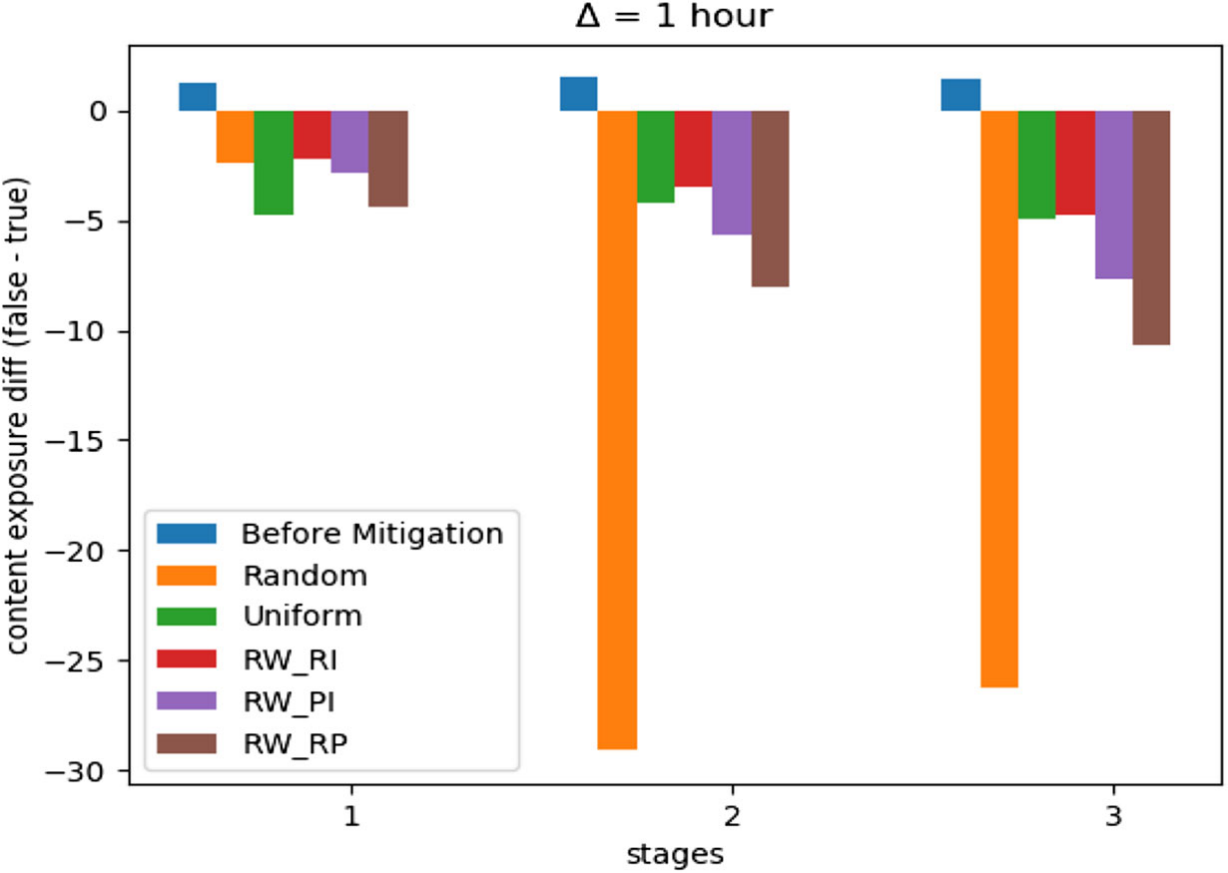
*Twitter16* mitigation performance on first three time stages, *C* = .05

**Fig. 9:**
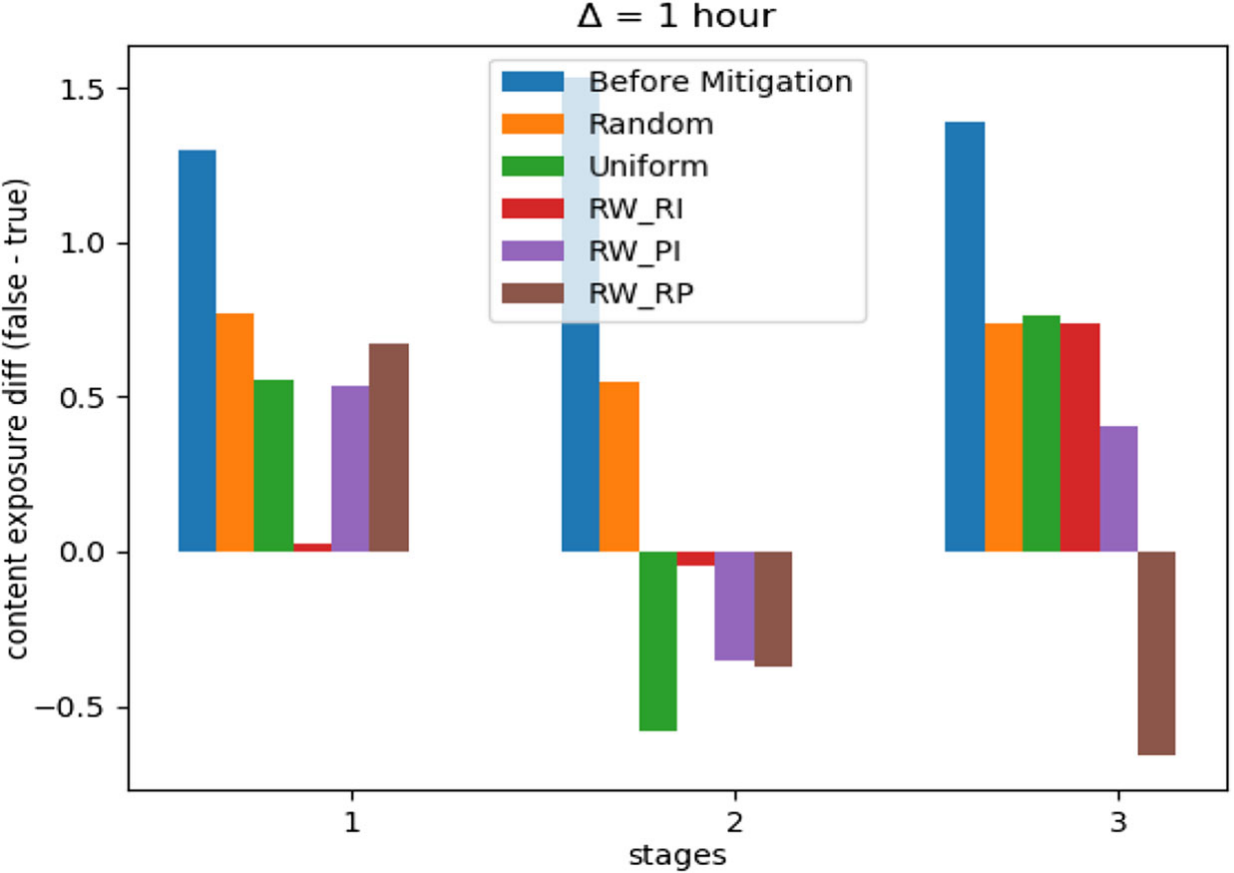
*Twitter16* mitigation performance on first three time stages, *C* = .0125

With a budget *C* = .05, Fig. 7 indicates how our optimization framework with the learning scheme *RW*_*RP*_ performed well with more stability, compared to other learning schemes in addition to another two baselines. However, the uniform distribution method performed approximately the same. Moreover, Fig. 8 shows how the random distribution outperformed all other methods for the next two time stages. We consider this as an interesting example of how *RW*_*RP*_ continued to be more robust compared to other LA-based learning schemes, but it failed to compete with both uniform and random strategies. However, a slight improve in the difference between *RW*_*RP*_ and the uniform and random distribution strategies can be noticed in Fig. 9, after repeating the experiment with only 25*%* of the original budget, where *C* = .0125. Therefore, our LA-based method showed more robustness when a more strict budget was used.

### *Twitter-COVID19*

**Fig. 10:**
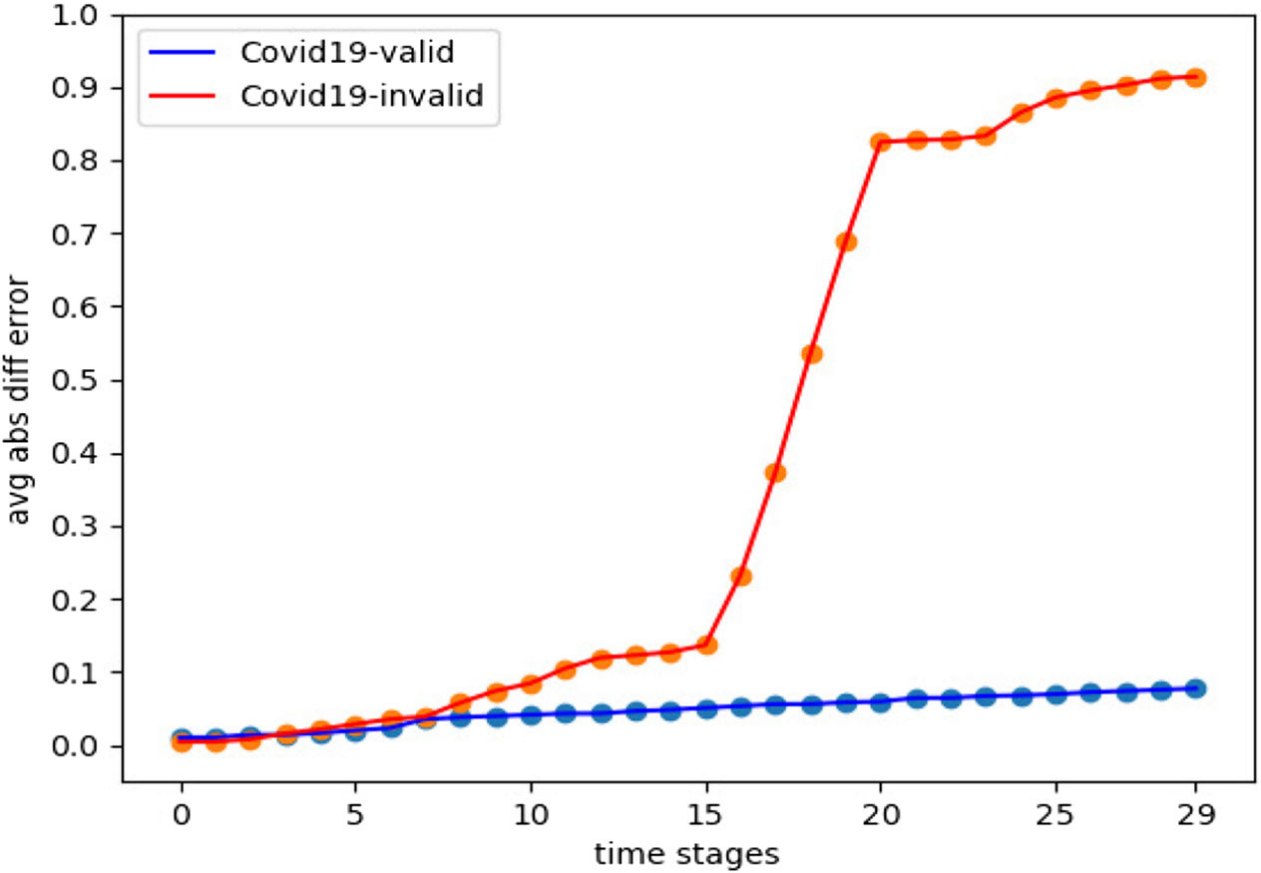
MHP simulation vs real data on scale of 1 less or more tweet per user difference on a time stage

As in *Twitter15* and *Twitter16*, the Hawkes simulation performance for *Twitter-COVID19* was measured according to Eq. 15. However, we observed more density in the events arrivals timestamps. Therefore, we set Δ = 10 minutes for a more convenient simulation. Figure 10 explains the Hawkes process performance on both irrational content and valid content, respectively.

The *Twitter-COVID19* dataset is also an interesting case for this study, since it has only one user who was spreading the undesired content and at the same time such user had the highest node centrality degree. That is, in such special situation, we would like to evaluate how our method was independent from the graph structure.

**Fig. 11:**
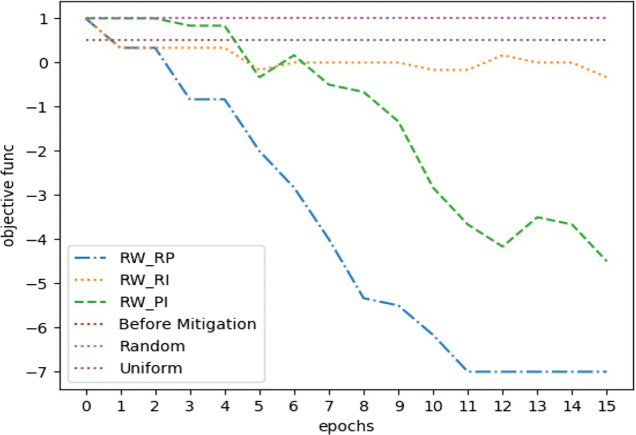
*Twitter-COVID19* mitigation performance on 1’st time stage, *C* = .05

For the simulation, we set the decay factors *w* = .7 and *w* = 1 for the irrational and rational content, respectively, while estimating such values following the same technique as in Goindani and Neville (2019). With a budget of *C* = .05, Fig. 11 shows how most of the LA-based methods outperformed the three baselines. However, it became obvious that *RW*_*RP*_ is the most reliable learning scheme for our optimization framework, since it converged earlier with better results in most of our experiments.

### Grid-search results

**Fig. 12:**
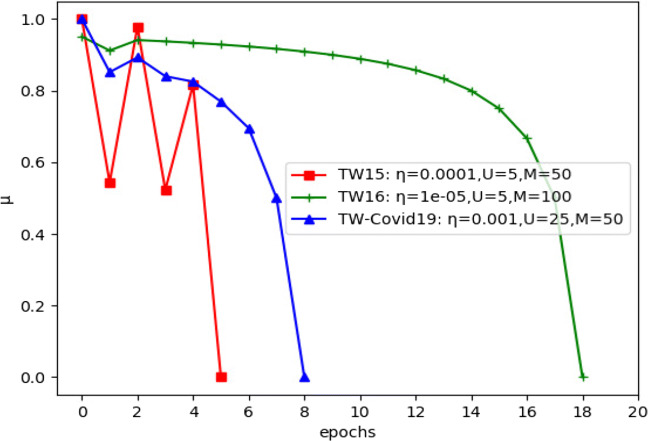
Convergence plot for the three datasets for *T* = *t*_0_,*C* = .05

Our method is dependant on three hyper parameters, the learning rate *η*, the random walk line (LA states) memory depth *M*, and the sample size |*U*^−^|. Therefore, we conducted a grid search to determine their best values. We evaluated the grid search results with respect to how different values of these parameters decreased the asymptotic error for convergence, and increased the risk reduction metric $\mathcal {K}$. The latter is discussed in details in Section 5. Figure 12 shows how we obtained a *Q-superlinear* convergence on the three datasets from the final estimated hyper parameters and the first MHP time stage and with budget *C* = .05. Tables 3, 4 and 5 gives a detailed explanation for the performance of different grid search hyper parameters values over all datasets for the first time stage and with budget *C* = .05. The selection criteria was mainly how much an acceptable risk reduction was achieved with the least possible epochs $\mathcal {I}$. Nevertheless, we considered values which had less effect on the computation speed, since even one epoch might be slower than another while using different values for |*U*^−^|, and *η*. That is because the number of calculations inside one epoch will increase when the network size increases. On the other hand, the learning rate parameter controls the density of the MHP generated events, since higher values of incentivization would lead to more generated counts, which would also increase the number of calculation steps. That is, we approached as much higher $\mathcal {K}$ and lower $\mathcal {I}$, while using as much lower values for |*U*^−^|, and *η*.

**Table 3:** *Twitter15* grid-search hyper parameters for *T* = *t*_0_,*C* = .05

$\mathcal {I}$	$\mathcal {K}$	|*U*^−^|	*η*	*M*
≈ 5	≈ [125*%*, 131*%*]	5	.0001	50
≈ 2	≈ [125*%*, 131*%*]	5	.0001	100
≈ 38	≈ [106*%*, 114*%*]	25	.0001	50
≈ 47	≈ [119*%*, 128*%*]	25	.0001	100
> 60	≈ [100*%*, 103*%*]	50	.0001	50
> 60	≈ [119*%*, 128*%*]	50	.0001	100
≈ 25	≈ [73*%*, 79*%*]	5	.00001	50
≈ 19	≈ [119*%*, 128*%*]	5	.00001	100
≈ 11	≈ [119*%*, 128*%*]	25	.00001	50
≈ 13	≈ [119*%*, 128*%*]	25	.00001	100
≈ 18	≈ [125*%*, 135*%*]	50	.00001	50
≈ 34	≈ [118*%*, 125*%*]	50	.00001	100
*N*.*A*	[0*%*, 0*%*]	5	.000001	50
*N*.*A*	[0*%*, 0*%*]	5	.000001	100
≈ 12	≈ [120*%*, 129*%*]	25	.000001	50
≈ 13	≈ [120*%*, 129*%*]	25	.000001	100
≈ 48	≈ [73*%*, 79*%*]	50	.000001	50
≈ 25	≈ [126*%*, 135*%*]	50	.000001	100

**Table 4:** *Twitter16* grid-search hyper parameters for *T* = *t*_0_,*C* = .05.

$\mathcal {I}$	$\mathcal {K}$	|*U*^−^|	*η*	*M*
≈ 4	≈ [227*%*, 241*%*]	5	.0001	50
≈ 5	≈ [235*%*, 253*%*]	5	.0001	100
≈ 30	≈ [235*%*, 253*%*]	25	.0001	50
≈ 30	≈ [119*%*, 128*%*]	25	.0001	100
> 60	≈ [190*%*, 209*%*]	50	.0001	50
> 60	≈ [184*%*, 200*%*]	50	.0001	100
≈ 10	≈ [219*%*, 235*%*]	5	.00001	50
≈ 19	≈ [250*%*, 276*%*]	5	.00001	100
≈ 12	≈ [168*%*, 183*%*]	25	.00001	50
≈ 12	≈ [242*%*, 265*%*]	25	.00001	100
≈ 50	≈ [506*%*, 550*%*]	50	.00001	50
≈ 57	≈ [242*%*, 265*%*]	50	.00001	100
≈ 24	≈ [243*%*, 266*%*]	5	.000001	50
> 60	≈ [242*%*, 265*%*]	5	.000001	100
≈ 24	≈ [242*%*, 265*%*]	25	.000001	50
≈ 13	≈ [239*%*, 260*%*]	25	.000001	100
≈ 48	≈ [138*%*, 150*%*]	50	.000001	50
≈ 18	≈ [199*%*, 210*%*]	50	.000001	100

**Table 5:** *Twitter-Covid19* grid-search hyper parameters for *T* = *t*_0_,*C* = .05

$\mathcal {I}$	$\mathcal {K}$	|*U*^−^|	*η*	*M*
≈ 21	≈ [330*%*, 340*%*]	5	.001	50
≈ 29	≈ [329*%*, 339*%*]	5	.001	100
≈ 10	≈ [330*%*, 340*%*]	25	.001	50
≈ 17	≈ [327*%*, 338*%*]	25	.001	100
≈ 15	≈ [331*%*, 342*%*]	50	.001	50
≈ 19	≈ [300*%*, 310*%*]	50	.001	100
*N*.*A*	[0*%*, 0*%*]	5	.0001	50
*N*.*A*	[0*%*, 0*%*]	5	.0001	100
*N*.*A*	[0*%*, 0*%*]	25	.0001	50
*N*.*A*	[0*%*, 0*%*]	25	.0001	100
*N*.*A*	[0*%*, 0*%*]	50	.0001	50
*N*.*A*	[0*%*, 0*%*]	50	.0001	100
*N*.*A*	[0*%*, 0*%*]	5	.00001	50
*N*.*A*	[0*%*, 0*%*]	5	.00001	100
*N*.*A*	[0*%*, 0*%*]	25	.00001	50
*N*.*A*	[0*%*, 0*%*]	25	.00001	100
*N*.*A*	[0*%*, 0*%*]	50	.00001	50
*N*.*A*	[0*%*, 0*%*]	50	.00001	100

The learning rate value had also some other effects on the results, for example, *Twitter-Covid19* dataset needed a higher value of *η* to make its users start to respond. That can be seen in Table 5, where $\mathcal {K} = 0$ in most of the experiments. Eventually, the memory depth parameter *M* was also essential to the computation, since it controls how fast the knapsack became full, which in turn, could affect the required number of epochs $\mathcal {I}$.

## Discussion

As discussed in Section 4, the random count range $N^{t}_{\mathcal {E}^{\pm }}$ defines an estimate range for the random variable *N*^*t*^, considering the random output and the average absolute difference error $\mathcal {E}$ that affects the mitigation metrics such as the difference between valid and invalid information. Therefore, the risk reduction percentage $\mathcal {K}$ can be calculated with regard to Eqs. 3 and 4 as per the one below. Where $\mathcal {E}$ is the sum of errors for *misinformation* and true events simulated counts, Table 6 shows the summed error for the datasets.

16 $$ \begin{array}{@{}rcl@{}} \mathcal{K}^{t_{s}}&=&\left[(1-\mathcal{E}) \cdot \left( \frac{\sqrt{[(F_{1} - T_{1}) - (F_{2} - T_{2})]^{2}}}{(F_{1} - T_{1})}\right) \cdot \left( \frac{1}{2}\right),\right.\\&& \left.\left( \frac{\sqrt{[(F_{1} - T_{1}) - (F_{2} - T_{2})]^{2}}}{(F_{1} - T_{1})}\right)\cdot \left( \frac{1}{2}\right)\right]^{t_{s}}, \end{array} $$

where *F*_1_ − *T*_1_≠ 0

**Table 6:** Datasets simulation accumulated error $\mathcal {E}$

	*t*_0_	*t*_1_	*t*_2_
*Twitter15*	.076	.076	.140
*Twitter16*	.083	.083	.163
*Twitter-COVID19*	.028	.028	.037

Where at a specific time stage *t*_*s*_, *F*_1_ − *T*_1_ represents the difference between false and true content before mitigation, and *F*_2_ − *T*_2_ is the difference after mitigation. Then, $\mathcal {K}^{t_{s}}$ indicates the estimates where such uncertain output would be, by measuring the distance between the two points (*F*_1_ − *T*_1_) , (*F*_2_ − *T*_2_) and creating a range between the weighted and the original calculation results. The estimated range values are divided by 2 in order to consider the output (*F*_2_ − *T*_2_ = 0) as only a 50*%* reduction, since there is still a 50*%* chance of being exposed to *misinformation* on the network. For cases when (*F*_1_ − *T*_1_ = 0), we omit it as the denominator, and we omit the division by 2 as well.

We have investigated how each learning scheme performed on the three datasets from the perspective of independence from network centrality measures. For instance, when *T* = *t*_0_, we obtained $\mathcal {P}(u_{influencer} \in U^{*} = 1)$, $\mathcal {P}(u_{influencer} \in U^{*} = .93)$, and $\mathcal {P}(u_{influencer} \in U^{*} = .71)$ for *Twitter15* for the three learning schemes *RW*_*RP*_, *RW*_*RI*_, and *RW*_*PI*_, respectively. On the other hand, for *Twitter16*, we got $\mathcal {P}(u_{influencer} \in U^{*} = .50)$, $\mathcal {P}(u_{influencer} \in U^{*} = 1)$, and $\mathcal {P}(u_{influencer} \in U^{*} = .57)$ for the three learning schemes *RW*_*RP*_, *RW*_*RI*_, and *RW*_*PI*_, when *T* = *t*_0_, respectively. While in *Twitter-Covid19*, we obtained $\mathcal {P}(u_{influencer} \in U^{*} = .19)$, $\mathcal {P}(u_{influencer} \in U^{*} = .20)$, and $\mathcal {P}(u_{influencer} \in U^{*} = .19)$ for the three learning schemes *RW*_*RP*_, *RW*_*RI*_, and *RW*_*PI*_, when *T* = *t*_0_, respectively. Since, the latter dataset is the case where the top influencer user had contributed only to *misinformation*, while other datasets had top influencers who contributed to true content, we would consider our method showing independence from graph structure when the top influencer user is not showing any potentials for circulating valid content.

### Evaluation

As mentioned earlier, we have adopted our own post-processed version of the *Twitter15* and *Twitter16* datasets. Further, it was unfeasible to apply all previous baseline mitigation methods on the same data samples we used. However, on the different datasets versions, Table 7 demonstrates by how far our LA-based method outperformed random and uniform budget distribution methods, with an analogy to previous reinforcement learning-based mitigation methods (MHP-U (Goindani and Neville 2019), V-MHP (Farajtabar et al. 2017), EXP (Farajtabar et al. 2017)). We refer to our method as LA-MHP, and the evaluation metric is the ratio between the correlation maximization $Y^{t_{s}}$ at a given time stage for each baseline and either random or uniform maximized correlation, when applied on the associated dataset version. Where the exposure amounts of both fake and true content are considered the correlation variable and constant, respectively ($Y^{t_{s}}=T^{t_{s}} F^{t_{s}}$). For instance, the ratio that indicates how LA-MHP performed against random distribution with regard to correlation maximization is calculated as $\frac {LA-MHP_{Y}}{RND_{Y}}$, where *Y* is calculated twice for both LA-MHP and RND over their MHP generated amount *T*. The results given in Table 7 proves how the LA-MHP model is competing with all baselines.

**Table 7:** Relative performance against random and uniform methods

Model	*Tw15-* RND	*Tw15-* UNIf	*Tw16-* RND	*Tw16-* UNIf
LA-MHP	2.37	2.11	2.35	1.71
MHP-U	2.06	1.93	3.20	1.80
V-MHP	1.54	1.87	2.80	1.50
EXP	1.33	1.21	2.04	1.12

## Conclusion

The emergence of the Multivariate Hawkes Processes (MHP) and their application on social media, have boosted the capabilities of social media analysis domain. Hence, MHP-based models became crucial to understand information diffusion and users actions prediction on social networks. Moreover, social media intervention-based approaches are highly depending on MHP to evaluate and improve the developed methods. MHP can be applied to mimic the users future behaviour on social networks after learning from some past actions on the network. Furthermore, MHP analyze the behaviour of users with regard to different factors. First, MHP can model an exogenous factor that causes a user to act. Then, the model takes into consideration the endogenous factors such as the network users historical behaviour. Therefore, the MHP construct powerful social media dynamics simulation models, where studying internal and external network motivations became possible and reliable.

Compared to deep reinforcement learning approaches, which were adapted in similar previous work, the explanation given in this study showed how our LA-based method is more reliable for a proactive *misinformation* mitigation strategy, since an LA is easier to understand and implement. Besides, our demonstrated method converged faster while using a notable smaller sample size, compared to the number of samples needed in similar previous work. Furthermore, and to the best of our knowledge, we were the first to apply LA for *misinformation* on social media.

Future work would investigate how politically biased users might not respond to a mitigation campaign, which will waste the incentivization budget. Furthermore, different objective functions should be investigated, for instance, in certain scenarios, we should consider fair mitigation for the influenced users, instead of calculating the average of differences between fake and true content, since an average for skewed individual differences distribution would not be enough to achieve optimum mitigation results.

## Footnotes

1 The term *misinformation* is sometimes replaced with *disinformation* in some literature. More conveniently, *misinformation* is the unintended spread of malicious content, while disinformation is purposely spreading malicious content. For the rest of this paper, we will use the term *misinformation* to refer to both phenomenons.

2 https://www.theguardian.com/world/2020/mar/24/coronavirus-cure-kills-man-after-trump-touts-chloroquine-phosphate
